# Analysis of genomic-length HBV sequences to determine genotype and subgenotype reference sequences

**DOI:** 10.1099/jgv.0.001387

**Published:** 2020-03-05

**Authors:** Anna L. McNaughton, Peter A. Revill, Margaret Littlejohn, Philippa C. Matthews, M. Azim Ansari

**Affiliations:** ^1^​ Nuffield Department of Medicine, Peter Medawar Building for Pathogen Research, South Parks Road, Oxford OX1 3SY, UK; ^2^​ Victorian Infectious Diseases Reference Laboratory, Royal Melbourne Hospital at the Peter Doherty Institute for Infection and Immunity, Melbourne, Australia; ^3^​ Department of Microbiology and Immunology, University of Melbourne, Melbourne, Australia; ^4^​ Department of Infectious Diseases and Microbiology, Oxford University Hospitals NHS Foundation Trust, John Radcliffe Hospital, Headley Way, Oxford OX3 9DU, UK; ^5^​ Oxford NIHR Biomedical Research Centre, John Radcliffe Hospital, Headley Way, Oxford OX3 9DU, UK; ^6^​ Wellcome Centre for Human Genetics, Roosevelt Drive, Oxford OX3 7BN, UK

**Keywords:** HBV, reference sequences, whole genome, phylogenetics

## Abstract

Hepatitis B virus (HBV) is a diverse, partially double-stranded DNA virus, with 9 genotypes (A–I), and a putative 10th genotype (J), characterized thus far. Given the broadening interest in HBV sequencing, there is an increasing requirement for a consistent, unified approach to HBV genotype and subgenotype classification. We set out to generate an updated resource of reference sequences using the diversity of all genomic-length HBV sequences available in public databases. We collated and aligned genomic-length HBV sequences from public databases and used maximum-likelihood phylogenetic analysis to identify genotype clusters. Within each genotype, we examined the phylogenetic support for currently defined subgenotypes, as well as identifying well-supported clades and deriving reference sequences for them. Based on the phylogenies generated, we present a comprehensive set of HBV reference sequences at the genotype and subgenotype level. All of the generated data, including the alignments, phylogenies and chosen reference sequences, are available online (https://doi.org/10.6084/m9.figshare.8851946) as a simple open-access resource.

## Introduction

Hepatitis B virus (HBV) is the prototype virus of the family *Hepadnaviridae*, an unusual family of partially double-stranded (ds) DNA viruses approximately 3.2 kb in length encoding four genes, including a reverse transcriptase, encoded in overlapping reading frames on a circular genome [1, 2]. To date, 9 genotypes of HBV have been characterized (A–I), along with a putative 10th genotype (J) [3]. The viruses display a relatively high amount of variation for a dsDNA virus [4], with the viral genotypes being further subdivided into upwards of 30 subgenotypes, many of which have distinct geographical and clinical associations [2].

At present, HBV genotyping is not being performed routinely in most clinical settings, as it has not been widely considered to be relevant to patient management. However, as more treatment data become available and improvements are made in patient-stratified clinical care, guidelines may change to reflect different genotype-specific recommendations. This has been exemplified by the management of hepatitis C virus (HCV) infection [5], and a similar approach for HBV is starting to emerge, with recent European Association for the Study of the Liver (EASL) guidelines for HBV treatment suggesting different stopping points for treatment non-response in genotypes A–D [6]. Evidence increasingly supports a role for HBV genotype in influencing disease progression, including risk of developing chronic infection, e-antigen seroconversion, transmission mode and the development of hepatocellular carcinoma [7, 8]. Studies often refer to ‘wild-type’ virus [8, 9], but wild-type for one genotype may not reflect consensus for other genotypes [10, 11]. Understanding the diverse range of HBV strains circulating globally, and their associations with disease, will allow us to move towards a more specific and precise approach to analysis.

A consistent approach to HBV subtyping is progressively more relevant, as interest in studying the associations between disease outcomes and viral sequence expands, and deep sequencing is used to investigate viral diversity in increasingly larger cohorts. A progressive body of sequencing data is emerging, and this expansion of available data is likely to increase over time (Fig. S1, available in the online version of this article); however, the number of sequences covering the full genome lags a long way behind sequences for single genes or shorter fragments. To date, numerous subtyping misclassifications have been documented for HBV, predominantly driven by the use of partial genome sequences rather than full-length genomes [9, 10], the inappropriate classification of recombinant strains [11] and publications redesignating same subgenotype classifications to distinctly different strains [12, 13]. Whilst a number of HBV sequence and analysis resources already exist, including HBVdb [14], HBVRegDB [15], HBVDR [16] and geno2pheno (https://hbv.geno2pheno.org/), each defines different sets of reference sequences, and frequently only at the genotype level. Criteria for the selection of reference sequences are often not provided with the resources, making it difficult to judge if the sequences are representative for the genotype. Several databases and resources offer subgenotyping from provided fasta sequences, but are not transparent about the sequences they use as references, making it unclear how many subgenotypes they can identify. Despite proposed criteria for defining HBV genotypes and subgenotypes [3], currently there is no single resource where a concise set of well-evaluated reference sequences and their designations are readily available for use in an easily accessible format.

With this dataset, we have set out to provide a unified resource, taking a pragmatic approach to describing the phylogeny of HBV genotypes A–I using all available genotyped sequences from the HBVdb repository [14]. We removed highly similar sequences from downloaded alignments and performed maximum-likelihood phylogenetic analysis. We used the phylogeny of whole-genome sequences to identify well-supported clades and selected representative reference sequences for each identifiable subgenotype.

## Methods

### Definitions

We based our analysis on previously agreed definitions regarding the classification of HBV sequences on the basis of their nucleotide sequence diversity [3], as follows.

- **Genotype:** nucleotide divergence of >7.5 % has been proposed as the threshold for the definition of distinct HBV genotypes [17].
- **Subgenotype:** Genotypes are further classified into subgenotypes, based on a divergence of 4–7.5 %, monophyletic clustering and strong bootstrap support for the clade [3].

In this study, we used strong bootstrap support (≥70) and monophyletic clustering to identify distinct clades and examined the pre-existing literature for the likely subgenotype designation. In instances when subgenotypes can be seen to split into distinct clades but genetic distance and poor bootstrap support do not suggest a unique subgenotype, such as in the case of subgenotypes A1 and C2, we have referred to these as ‘A1 (1)’ and ‘A1 (2)’.

### HBV genome numbering convention

As HBV is a circular virus, there is technically no ‘start’ or ‘end’ to the genome. We have followed the convention in the field (and HBVdb) for this study, defining nucleotide (nt)1 at an *EcoR1* restriction site in the Pol/Surface overlap (GAA/TTC, with nt1 starting at the first T) [2]. The *EcoR1* site is hypothetical in many HBV sequences, with GAA/CTC being more frequent in many genotypes.

**Fig. 1.**
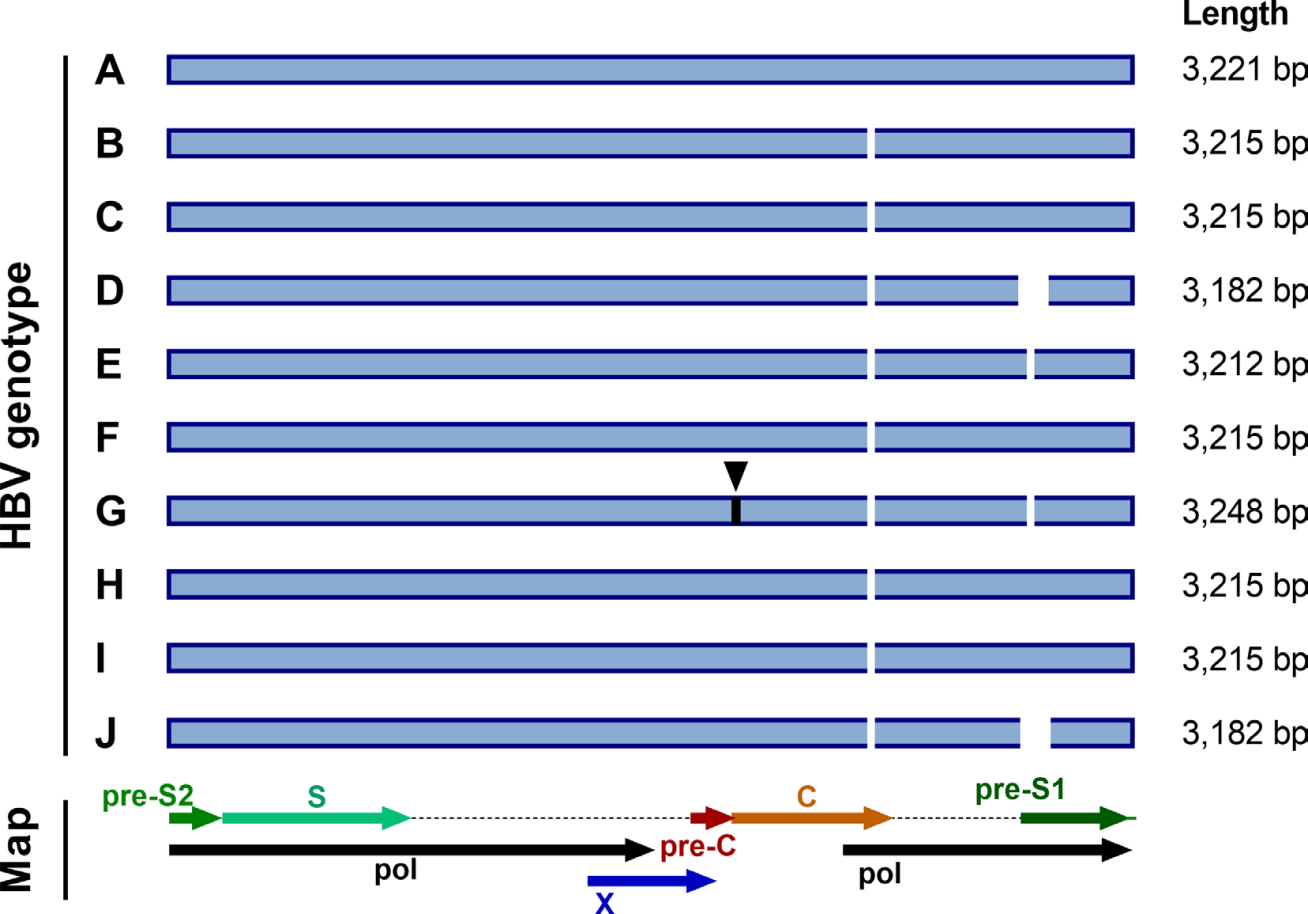
HBV genome lengths of each genotype. Standard genome lengths, and sites with deletions and insertions, are illustrated for genotypes A–J, along with a map of the HBV genome layout. Deletions and insertions are shown relative to genotype A, which is widely used as a numbering reference for HBV. Deletions are shown as white gaps and sites of insertions are indicated in black with triangles above them. All genotypes have a 6 bp deletion in the core (C), relative to genotype A (at nucleotide (nt)2354). Genotypes E and G have a 3 bp deletion in the pre-S1 region (at nt2861) and genotypes D and J have a 33 bp deletion at the start of the pre-S1 region (at nt2854) [2]. Genotype G also has a 33 bp insertion in the core (at nt1903).

Deletions and insertions are relatively common in HBV sequences [18, 19], meaning that a standardized approach to numbering is also required. We have followed convention and used the genotype A strain X02763 [20] as a numbering reference. Genotype A sequences are widely used as references as they are 3221 bp in length, with all other genotypes having a 6 bp deletion in the core gene (Fig. 1). Genotypes D, E, G and J have additional characteristic deletions in the pre-S1 region (Fig. 1). All deletions should be described relative to genotype A, noting the nucleotide numbers that are missing. Insertions should also give the site of insertion relative to the genotype A reference, and use a decimal point to indicate the length of the insertion. For example, the characteristic 33 bp insertion seen in genotype G at nucleotide 1903 should be described as 1903.33.

### Sources of sequence data

We downloaded all available genomic-length genotyped HBV sequences (*n*=7108) for genotypes A–H from HBVdb [14] on 31 January 2019, and removed sequences indicated as recombinant by the database (*n*=767), giving a total of 6341 sequences. We used HBVdb as the database runs sequences downloaded from Genbank through a genotyping algorithm prior to inclusion and all listed sequences are annotated to ensure that they follow the HBV genome numbering [14], as outlined above.

We enhanced our dataset by excluding two sequences from the genotype G phylogeny, as they had been incorrectly genotyped with the HBVdb algorithm. Both sequences (FJ023674 and FR714503) were found to be partial genotype C recombinants. We also identified an additional 71 sequences belonging to genotype I and to genotype C and D subgenotypes (under-represented in the HBVdb database) on GenBank and added these to our dataset, giving a total of 6412 sequences. Ancient HBV sequences, isolated from bodies hundreds of years old [21, 22], are present in these databases, and they were not excluded from this analysis, unless they grouped distantly from a known genotype.

### Existing reference sequences

We included the genotype A strain X02763 [20] in the alignment with other reference sequences as this sequence (length 3221 bp) is widely used as a numbering reference. Only genotype G has insertions relative to genotype A (Fig. 1). We suggest that the current NCBI HBV reference sequence, NC_003977.2 [23], a genotype D isolate, is less suitable as a numbering reference, as genotype D strains have a 33 bp deletion in the pre-S1 region, making them the shortest length HBV genotype at 3182 bp (Fig. 1). Nonetheless, we also included NC_003977.2 in the reference alignment, as it is frequently used as a reference.

### Phylogenetic analysis

**Fig. 2.**
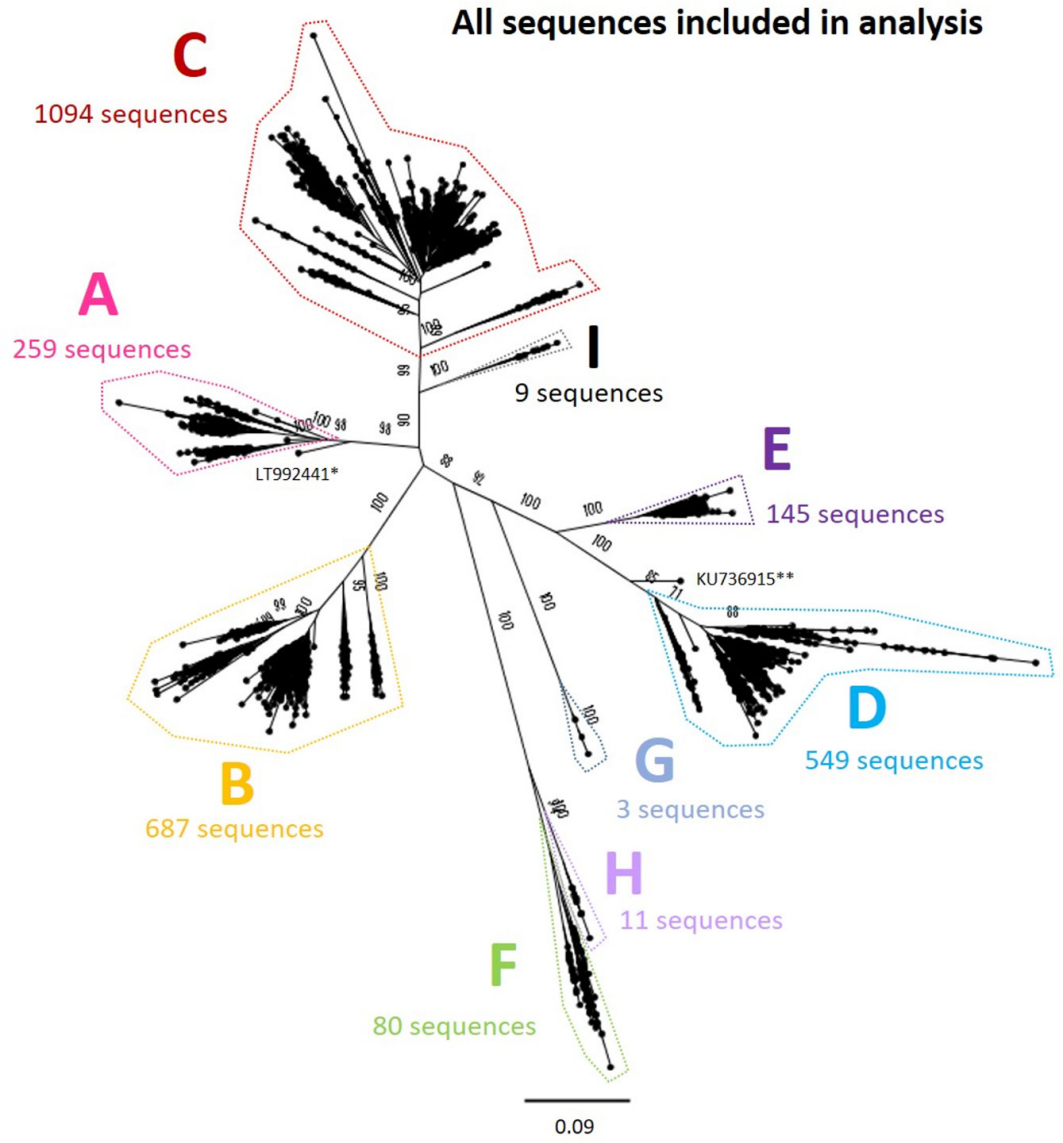
Genomic-length maximum-likelihood phylogeny of all genotype A–I HBV sequences included in analysis (*n*=2839) after removing highly similar sequences, indicating the number of sequences in each genotype analysed separately (Figs 3–10). Bootstrap support ≥70 after 1000 replicates is given for the deepest branches on the tree. The scale bar indicates the estimated nucleotide substitutions per site. *, a strain known to be from a 14th century skeleton clustering distantly with genotype A, LT992441, was removed from the subsequent analysis. **, KU736915 was identified as a genotype D/E recombinant and removed from the subsequent analysis.

We aligned all sequences using mafft [24] and calculated pairwise distances between all isolates. In order to reduce the possibility that multiple sequences from a single individual or closely related cluster could dominate the phylogeny, we kept only one sequence from each group of identical sequences, and used hierarchical clustering to group isolates based on their pairwise distance. We identified clusters of sequences that were within ≤1 % of each other, retaining a single sequence from each cluster for the analysis (retaining the sequence with the lowest proportion of ambiguous sites). After stripping out these closely related sequences, we generated a maximum-likelihood phylogenetic tree of the remaining 2839 sequences using RAxML [25], including 1000 bootstrap replicates (Fig. 2). We used this tree to manually delineate sequences belonging to each genotype and excluded any remaining recombinant sequences that had not been identified earlier in the process.

**Fig. 3.**
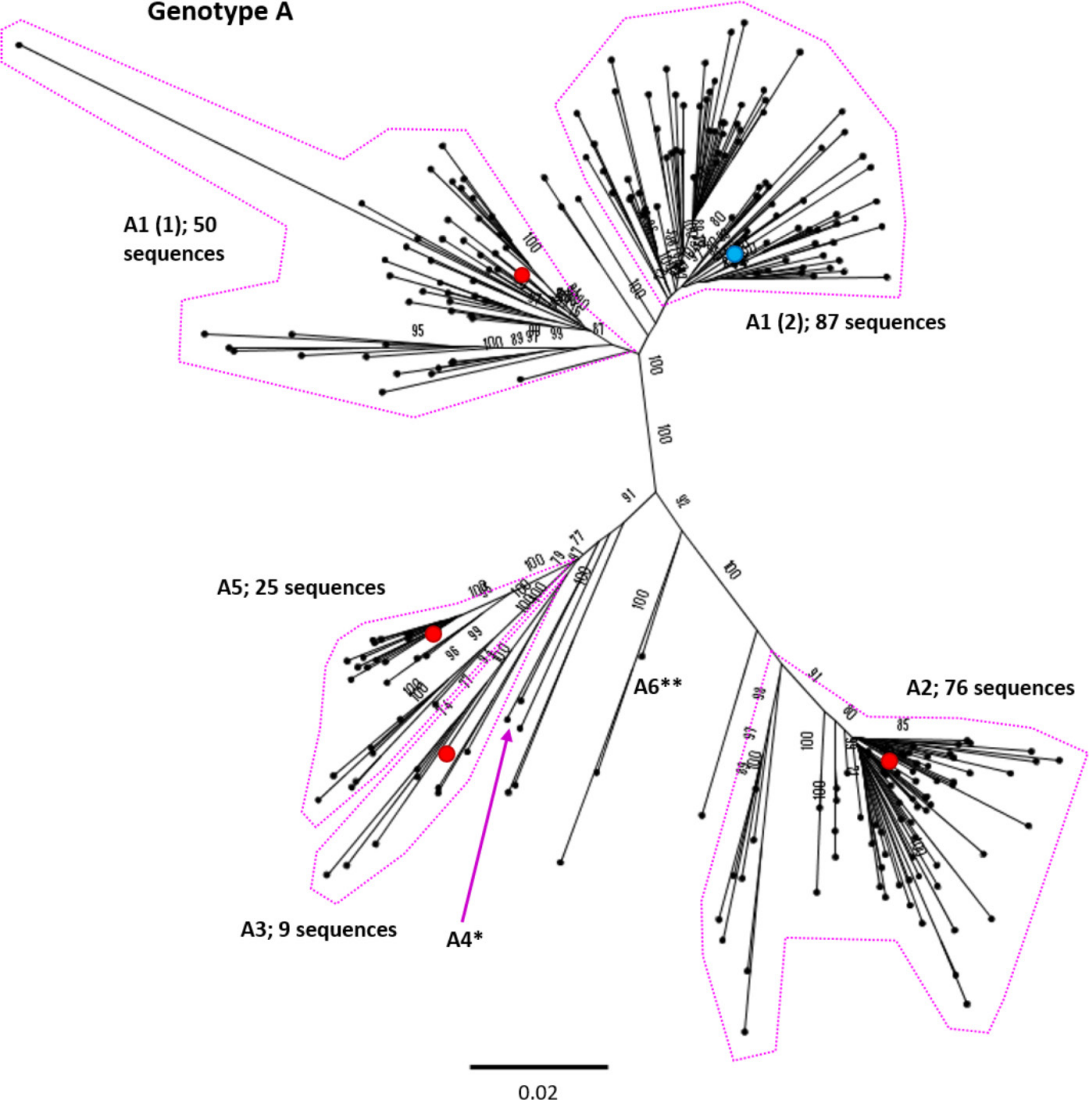
Genomic-length maximum-likelihood phylogeny of HBV genotype A sequences (*n*=259). Well-defined clades have been highlighted with coloured dotted lines and reference sequences for each clade indicated (red dots). The proposed reference strain for the genotype, FJ692557, is highlighted with a blue dot. The subgenotype is given where it could be reliably identified. Bootstrap support for branches ≥70 after 1000 replicates is indicated. The scale bar indicates the estimated nucleotide substitutions per site. Previous work has confirmed that there are at least five genotype A subgenotypes, although debate continues about whether A3, A4 and A5 should all be considered to be ‘quasi-subgenotype A3’ [9]. Few sequences for subgenotypes *A4 (KM606737) and **A6 (GQ331046) were retained in the study after pairwise analysis. The putative subgenotype A6 has previously been identified in three African-Belgian patients [37].

**Fig. 4.**
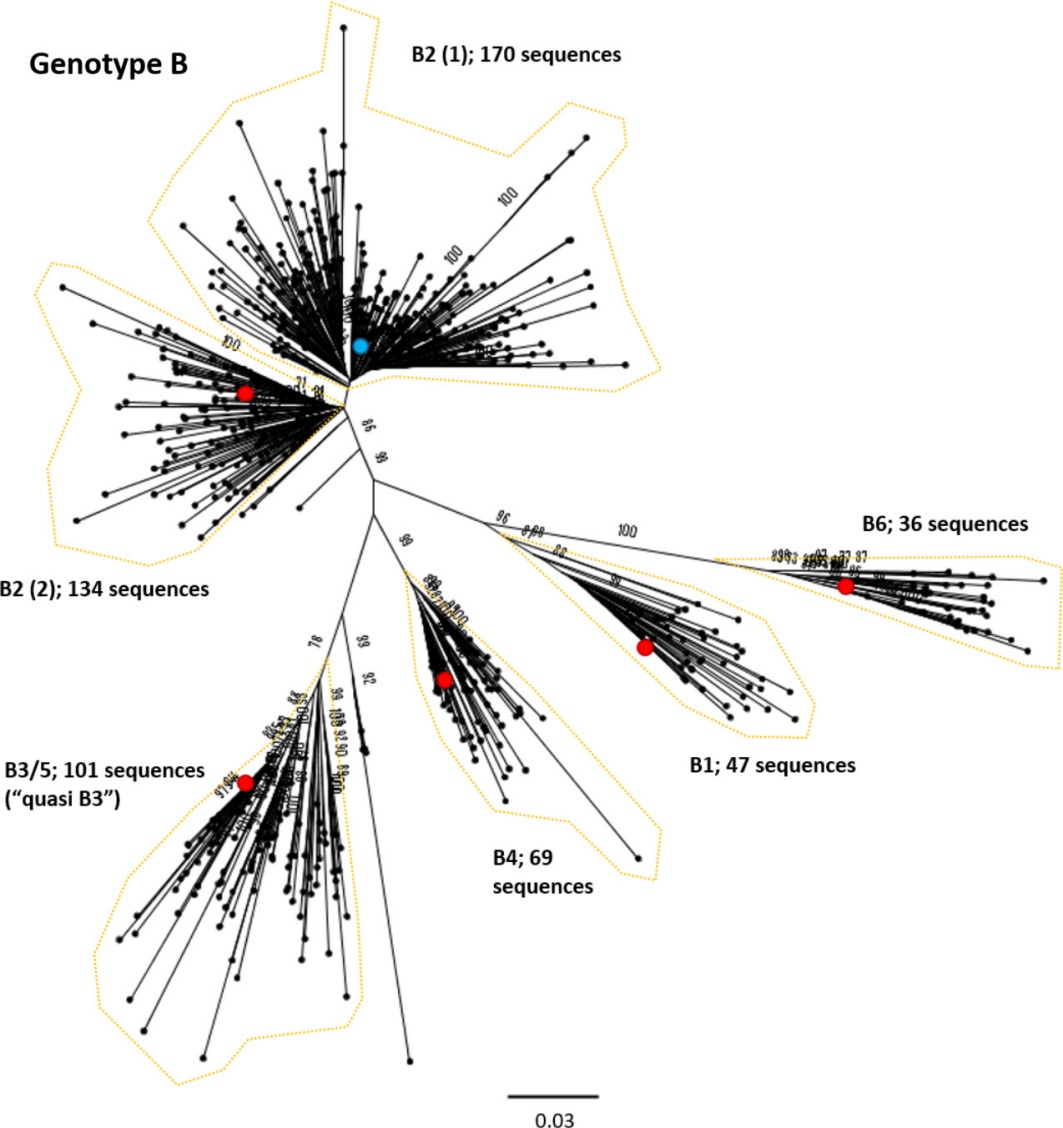
Genomic-length maximum-likelihood phylogeny of HBV genotype B sequences. Well-defined clades have been highlighted with coloured dotted lines and reference sequences for each clade indicated (red dots). The proposed reference strain for the genotype, GU815637, is highlighted with a blue dot. The subgenotype is given where it could be reliably identified. Bootstrap support for branches ≥70 after 1000 replicates is indicated. The scale bar indicates the estimated nucleotide substitutions per site. An evaluation of the genotype B phylogeny reclassified a number of putative subgenotypes as quasi-B3, with debate continuing on whether or not this should also include B5 [53].

**Fig. 5.**
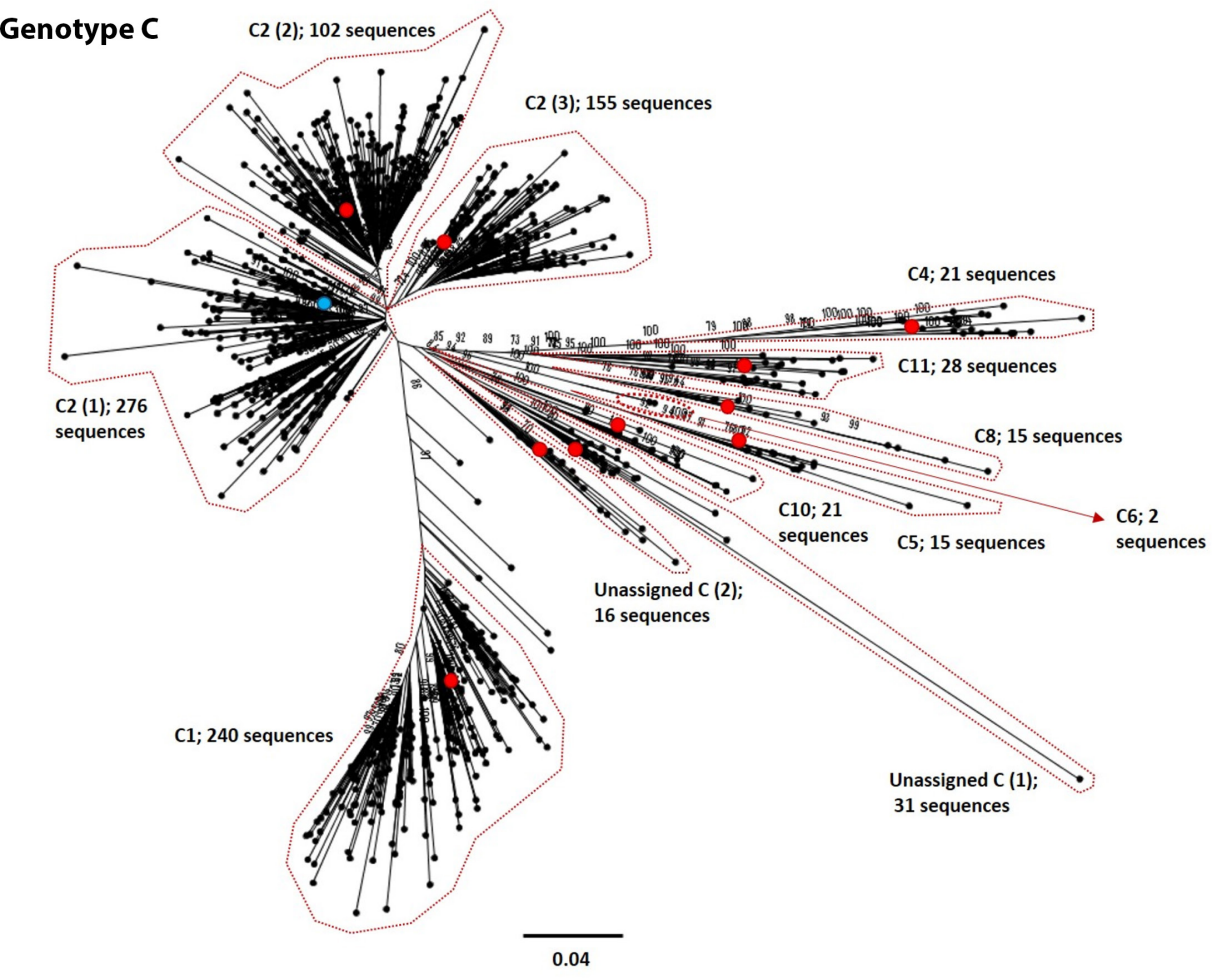
Genomic-length maximum-likelihood phylogeny of HBV genotype C sequences. Well-defined clades have been highlighted with coloured dotted lines and reference sequences for each clade indicated (red dots). The proposed reference strain for the genotype, GQ377617, is highlighted with a blue dot. The subgenotype is given where it could be reliably identified. Bootstrap support for branches ≥70 after 1000 replicates is indicated. The scale bar indicates the estimated nucleotide substitutions per site. We were unable to verify the subgenotype of two genotype C clades, and these have been designated unassigned clades 1 and 2 [unassigned_C (1) and unassigned_C (2), respectively].

**Fig. 6.**
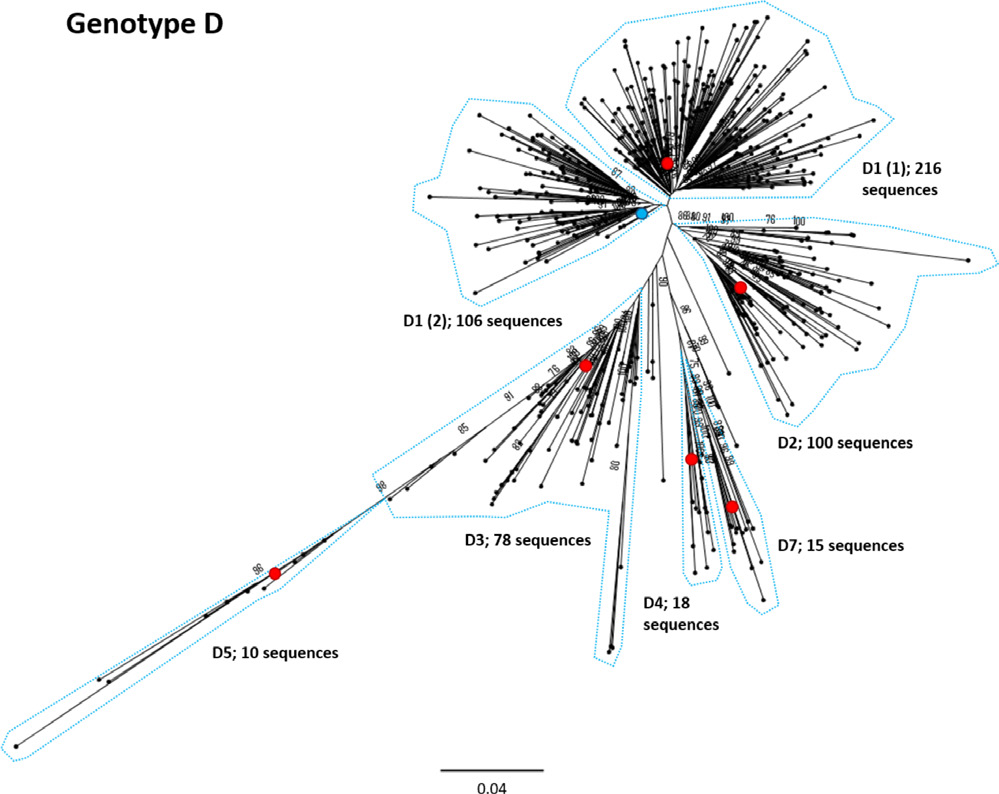
Genomic-length maximum-likelihood phylogeny of HBV genotype D sequences. Well-defined clades have been highlighted with coloured dotted lines and reference sequences for each clade indicated (red dots). The proposed reference strain for the genotype, KC875277, is highlighted with a blue dot. The subgenotype is given where it could be reliably identified. Bootstrap support for branches ≥70 after 1000 replicates is indicated. The scale bar indicates the estimated nucleotide substitutions per site. Previous work has indicated that the D3 and D6 strains cluster together and should be classed as a single subgenotype [54].

**Fig. 7.**
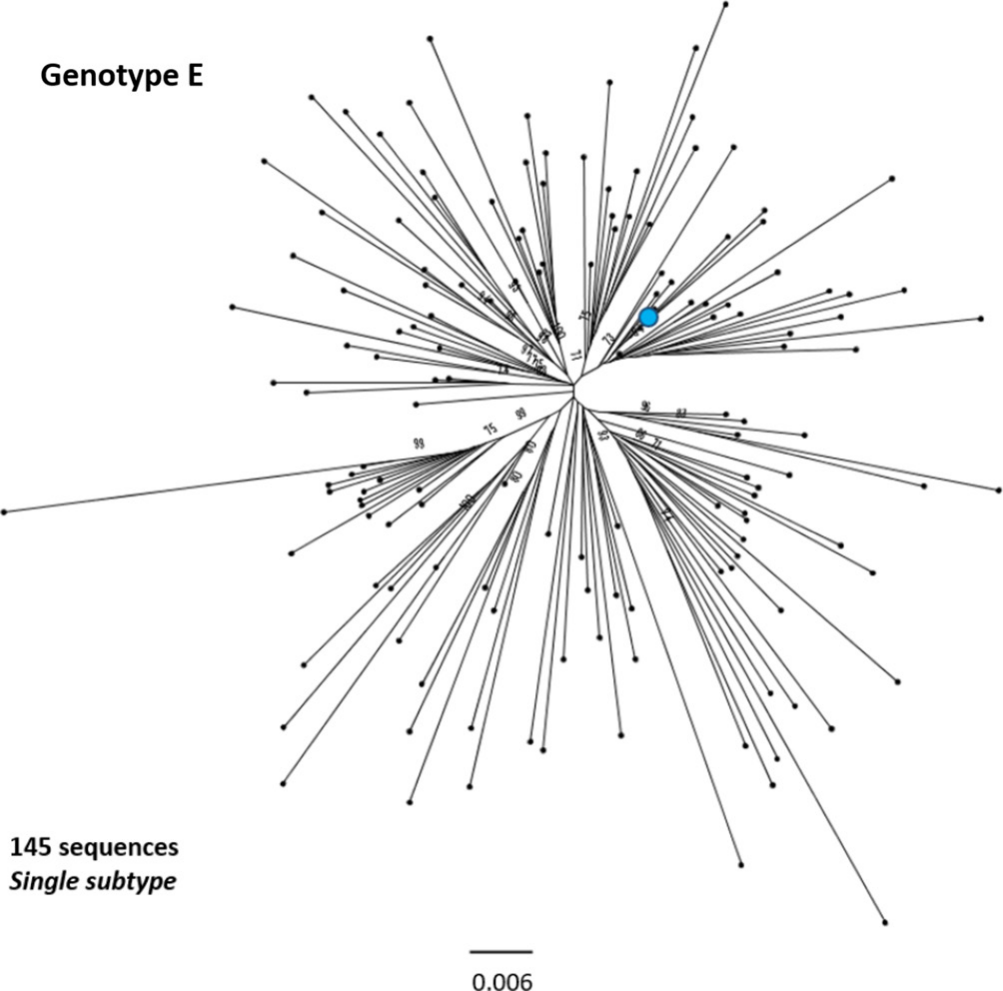
Genomic-length maximum-likelihood phylogeny of HBV genotype E sequences. Genotype E sequences do not diverge into distinct subgenotypes. Bootstrap support for branches ≥70 after 1000 replicates is indicated. The scale bar indicates the estimated nucleotide substitutions per site. The proposed reference strain for the genotype, GQ161817, is highlighted with a blue dot.

**Fig. 8.**
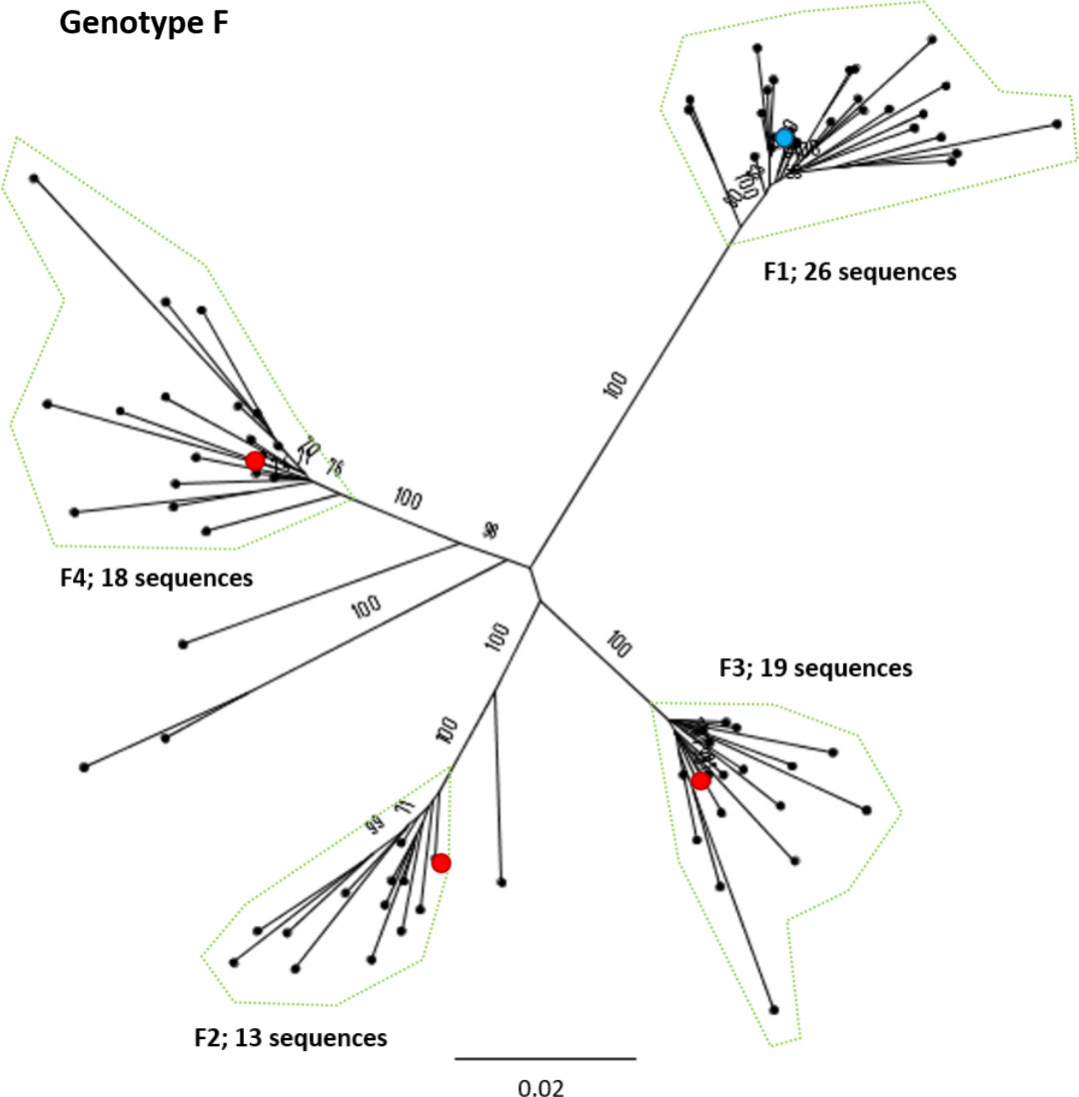
Genomic-length maximum-likelihood phylogeny of HBV genotype F sequences. Well-defined clades have been highlighted with coloured dotted lines and reference sequences for each clade indicated (red dots). The proposed reference strain for the genotype, HM585194, is highlighted with a blue dot. The subgenotype is given where it could be reliably identified. Bootstrap support ≥70 after 1000 replicates is given. The scale bar indicates the estimated nucleotide substitutions per site.

**Fig. 9.**
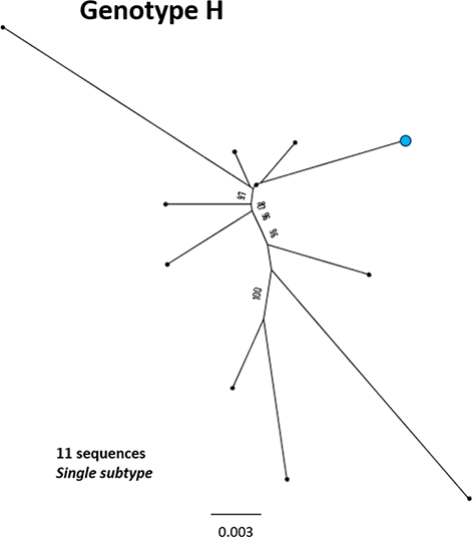
Genomic-length maximum-likelihood phylogeny of HBV genotype H sequences. Genotype H sequences do not diverge into distinct subgenotypes. Bootstrap support ≥70 after 1000 replicates is given. The proposed reference strain for the genotype, FJ356715, is highlighted with a blue dot. The scale bar indicates the estimated nucleotide substitutions per site.

**Fig. 10.**
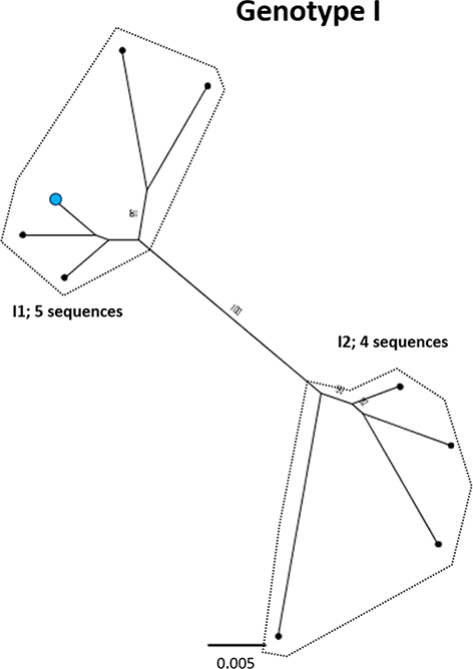
Genomic-length maximum-likelihood phylogeny of HBV genotype I sequences. Well-defined clades have been highlighted with coloured dotted lines and reference sequences for each clade indicated (red dots). The proposed reference strain for the genotype, AB562463, is highlighted with a blue dot. Bootstrap support for branches ≥70 after 1000 replicates is indicated. The scale bar indicates the estimated nucleotide substitutions per site.

We aligned the sequences for each genotype separately and generated maximum-likelihood phylogenetic trees as previously described. We examined the phylogenetic trees and generated bootstrap values to define distinct clades containing ≥2 sequences with strong phylogenetic support (Figs 3–10). Phylogenetic trees were examined in both traditional and radial layouts to ensure that the subgenotypes and clades were being delineated accurately. We excluded genotype G from the phylogenetic analysis, as after removing highly similar sequences there were just three sequences remaining.

### Definition of new reference sequences

For each well-defined clade, we generated a consensus sequence and selected the closest clinical isolate to the consensus sequence as the reference strain for that clade. In instances where well-represented subgenotypes (with >100 sequences present in the analysis) clearly diverged into multiple distinct clades, we provided multiple reference sequences for the subgenotype, representing each clade. We applied a process of quality assurance to the selection of reference strains as follows.

- We verified that isolates selected as reference strains were associated with an associated publication indexed in PubMed.
- We checked that reference strains were genuine clinical isolates (rather than *in silico* reconstructions such as consensus sequences).
- We rejected isolates that had insertions or deletions relative to the majority of sequences in the clade.
- We avoided (where possible) sequences with many ambiguous sites.
- We avoided use of sequences containing known drug resistance mutations as reference strains. Thus in the situation in which a sequence containing an rtM204 substitution in the ‘YMDD’ motif (associated with escape from lamivudine therapy [26]) was initially selected as a reference sequence, and the resistance-associated mutation was not occurring in other sequences in the clade, we rejected this and selected the next closest sequence to consensus.
- We selected three subgenotype references with Hamming distances >50 relative to the consensus sequence for the subgenotype clade (subgenotypes C4, C11 and genotype C UA (2). More closely related sequences were present within the subgenotype clade but indels in the sequences and a lack of supportive publications meant that these sequences were not selected.

To define genotype reference sequences, we selected the reference sequence for the subgenotype that contributed the largest number of sequences to that genotype (Table 1, grey boxes). In cases where there were multiple clades for the subgenotype, we selected the sequence from the one of the largest clades of the most well-represented subgenotype as the reference. Where there was strong evidence to support specific subgenotypes from both the literature and the phylogenetic analysis, we included the subgenotype in the analysis. Where subgenotype designation was unclear, we used blast to compare the sequence(s) in the clade to other sequences in online databases to see if the subgenotype could be identified. We also examined trees to find the accession numbers of subgenotypes that did not have an easily identifiable clade in the analysis using isolates listed in GenBank. If they were not present, these subgenotypes were most likely removed during the pairwise analysis for being highly similar to sequences already in the analysis, suggesting further examination of their designation as a unique subgenotype may be warranted. In situations in which we could not ascertain which subgenotype a clade belongs to, despite examination of GenBank records and blast analysis of sequences, we described these as ‘unassigned clades’ (both in genotype C, UA (1) and UA (2), Fig. 5).

**Table 1.** Proposed reference sequences for HBV genotypes, subgenotypes and clades The number of sequences in each clade is given for each subgenotype and clade identified. Note that the total sum of the subgenotype sequence clusters may not correspond to the total number of genotype sequences, as a number of sequences did not group within a specific clade. Reference sequences for the genotypes are highlighted in grey boxes. In Figs 3–10, genotype references are marked with blue dots and subgenotype reference sequences are marked with red dots. Subgenotypes B5, C7, C9 and D6 are not included, as these sequences either did not cluster as monophyletic clades or were not retained in our analysis. Hamming distance indicates the number of nucleotide differences between the clade consensus and the chosen reference. The pairwise distance is the number of nucleotide differences between the clade consensus and the chosen reference normalized by length of the genome.

HBV genotype	Subgenotype	No. of sequences	Reference GenBank ID	Hamming distance	Pairwise distance	References	Collection year*	Country of origin
A	A1 (1)	50	KP168423	26	0.008	[55]	2012	Kenya
	**A1 (2)**	**87**	**FJ692557**	21	0.007	[56]	2006	Haiti
	A2	80	EU594385	5	0.002	[57]	2004	Estonia
	A3	9	AM184126	29	0.009	[58]	2005	Gabon
	A4	2	KM606737	n/a	n/a	[59]	2015	Cuba
	A5	25	FJ692601	10	0.003	[60]	2006	Haiti
	A6	2	GQ331046	n/a	n/a	[37]	2006	Belgium
B	B1	47	D23679	23	0.007	[61]	1993	Japan
	B2 (1)	131	JQ801514	15	0.005	[62]	2009	Thailand
	**B2 (2**)	**294**	**GU815637**	10	0.003	[63]	2010	Taiwan, ROC
	B3	106	AP011085	23	0.007	[64]	2001	Indonesia
	B4	69	AB073835	35	0.011	[65]	2001	Japan
	B6	36	AB287314	29	0.009	[66]	2006	Alaska
C	C1†	240	DQ089781	29	0.009	[67]	2005	Hong Kong SAR
	C2 (1)	261	KC774182	21	0.007	[68]	2012	PR China
	**C2 (2**)	**280**	**GQ377617**	14	0.004	[69]	2007	PR China
	C2 (3)	157	AP011098	10	0.003	[70]	2009	Indonesia
	C4	21	KF873526	68	0.021	[71]	2011	Australia
	C5	15	AP011099	41	0.013	[70]	2009	Indonesia
	C6‡	2	EU670263	28	0.009	[29]	2008	Philippines
	C8	15	AP011107	42	0.013	[70]	2009	Indonesia
	C10	21	KJ173333	33	0.010	[72]	2012	PR China
	C11§	28	AB554015	86	0.027	[28]	2010	Indonesia
	UA (1)	31	KC774298	15	0.005	[68]	2012	PR China
	UA (2)	16	DQ089802	55	0.018	[67]	2005	Hong Kong SAR
D	D1 (1)	216	AB222711	11	0.003	[73]	2005	Uzbekistan
	**D1 (2**)	**106**	**KC875277**	17	0.005	[74]	2013	India
	D2	100	MF925358	21	0.007	[75]	2015	Bangladesh
	D3	78	FJ692507	18	0.006	[60]	2006	Haiti
	D4	15	FJ692533	17	0.008	[60]	2006	Haiti
	D5	15	GQ205389	19	0.006	[76]	2008	India
	D7	15	FJ904435	44	0.014	[77]	2006	Tunisia
E	**n/a**	**145**	**GQ161817**	19	0.006	[78]	2006	Guinea
F	**F1**	**26**	**HM585194**	13	0.004	[79]	2007	Chile
	F2	13	DQ899143	26	0.008	[80]	2006	Venezuela
	F3	19	MH051986	17	0.005	[81]	2011	Venezuela
	F4	18	KJ843175	17	0.005	[82]	2012	Argentina
G	**n/a**	**3**	**AB056513**	n/a	n/a	[83]	2001	USA
H	**n/a**	**11**	**FJ356715**	36	0.011	[84]	2008	Argentina
I	**I1**	**5**	**AB562463**	17	0.005	[85]	2007	Vietnam
	I2	4	FJ023669	17	0.005	[86]	2008	Laos
J¶	**n/a**	**1**	**AB486012**	n/a	n/a	[86]	2006	Japan/Borneo

n/a, not applicable; (Subgenotype) this genotype does not diverge into multiple subtypes; (Hamming/Pairwise distance), too few sequences identified belonging to the genotype/subgenotype to generate consensus sequences for selection of closest biological isolate.

*Collection date of sample or year submitted to GenBank (if collection date not given).

†C1 is a large clade that also contains sequences labelled as subgenotype C3, with no clear separation between the two sets of sequences.

‡This sequence has been used previously as a subgenotype C7 reference in a number of publications [21, 22]. No other putative C7 sequences are proposed in the literature.

§C11, a large number of sequences were unpublished in this clade (>30 first closest seqs).

¶Genotype J remains putative, with a single isolate identified in a Japanese patient. The isolate shows considerable divergence from other known HBV strains and is thought to be a recombinant of genotype C and a gibbon HBV isolate.

## Results

### Generation of a dataset of genomic-length HBV sequences

We downloaded a total of 7108 full-length HBV sequences from the hepatitis B virus database (HBVdb, https://hbvdb.ibcp.fr/HBVdb/) on 31 January 2019. Following clean-up of the dataset to remove identical sequences and recombinants (as described in detail in the Methods section) we were left with a total of 5972 sequences. We aligned the sequences and calculated pairwise distances for hierarchical clustering to remove highly similar isolates (see the Methods section). At this stage we were left with 2839 sequences on which we based our phylogenetic analysis (all sequences are shown in Fig. 2).

### Classification of HBV genotypes and subgenotypes and designation of references

We selected appropriate reference sequences to represent each genotype and subgenotype groups; these sequences are shown in the context of the overall phylogeny highlighted in Figs 3–10 and listed in Table 1. In cases where we could identify multiple distinct clades within a single subgenotype (subgenotypes A1, B2, C2, D1; Figs 3–6), we selected a reference for each clade to ensure that the diversity within the subgenotype was represented. In genotypes A–D, the most well-represented subgenotypes contained ≥45 % of all the sequences within that genotype. Sequences were most evenly distributed between subgenotypes in genotype F, with the commonest subgenotype accounting for 33 % of sequences.

### Ambiguities and inconsistencies in existing data

There are a number of poorly characterized and disputed subgenotypes in the literature, particularly within genotype C, making accurate identification of the subgenotypes challenging. Strong evidence to support a number of previously described clades was also difficult to obtain, and there were specific difficulties in the assessment of certain subgenotypes; D6, C7 and C9 were not easily distinguishable in this analysis. A search of GenBank indicated a single genomic-length sequence of subgenotypes A4 (KM606737), A6 (GQ331046) and D6 (KF170740) in the database, and no evidence of genomic-length sequences for subgenotype C7 or C9 isolates. Both A4 and A6 isolates clustered away from other sequences in the analysis, but few additional isolates of the subgenotypes were retained after the removal of highly similar sequences. In the case of subgenotype C7, previous publications on the phylogeny of genotype C used EU670263 as a C7 reference strain [27, 28]. However, in GenBank EU670263 is listed as subgenotype C6 [29] and clusters within our phylogenetic analysis with GU721029, which is also designated subgenotype C6. A single provisional isolate of subgenotype C9 exists (AP011108), having been proposed in 2010 [27], but this designation is not present in the GenBank data associated with the sequence. Likewise, subgenotypes C13–16 have been described [30], but we were unable to distinguish these as distinct subgenotypes in our analysis. The D6 isolate KF170740 was not retained in the sequences we selected for analysis, suggesting that it is closely related to another genotype D subgenotype.

### Comparative phylogenies and pairwise distance of HBV genotypes

**Fig. 11.**
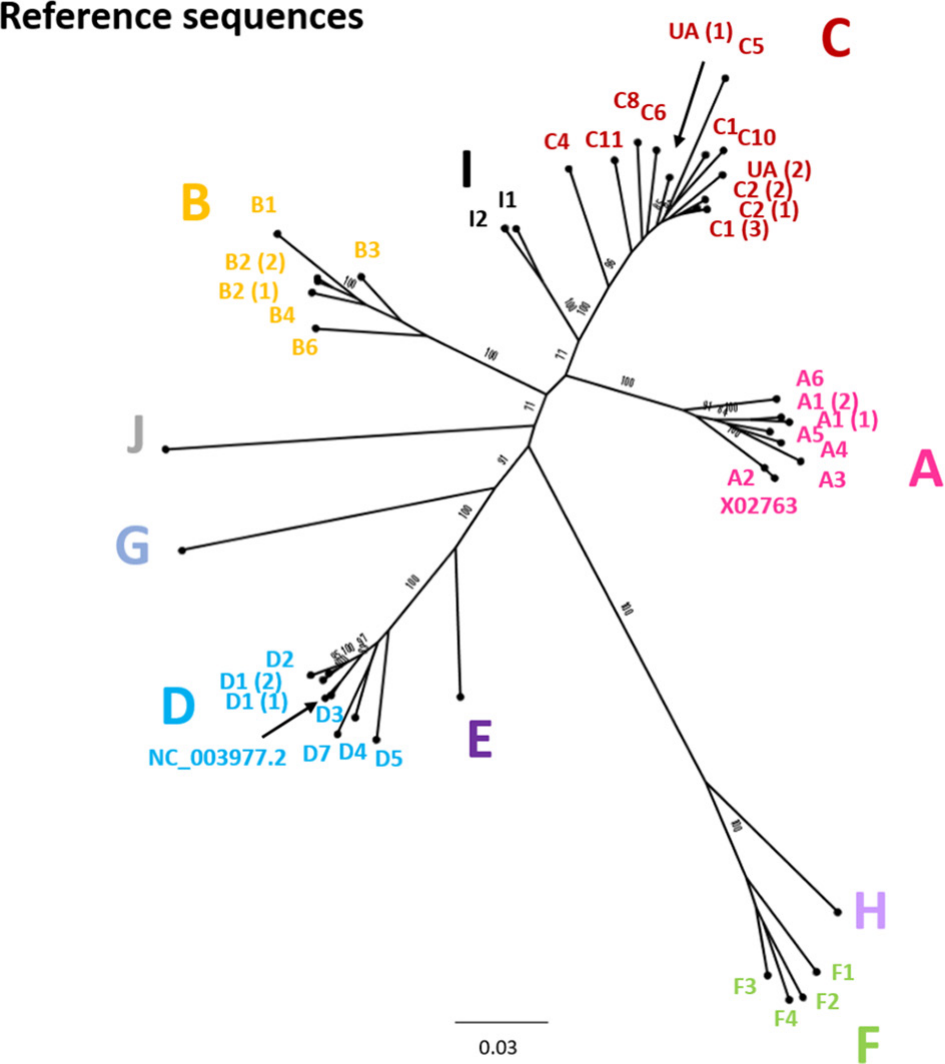
Genomic-length maximum-likelihood phylogenetic tree of HBV genotype, subgenotype and clade reference strains identified in Figs 3–10 and listed in Table 1 with accession numbers. The genotype is given in each case and the subgenotype or clade identification is given where possible. Bootstrap support for branches ≥70 after 1000 replicates is indicated. The scale bar indicates the estimated nucleotide substitutions per site. In addition to the references identified in Figs 3–10, genotype A isolate X02763 and genotype D isolate NC_003977.2 have been included in the tree.

**Fig. 12.**
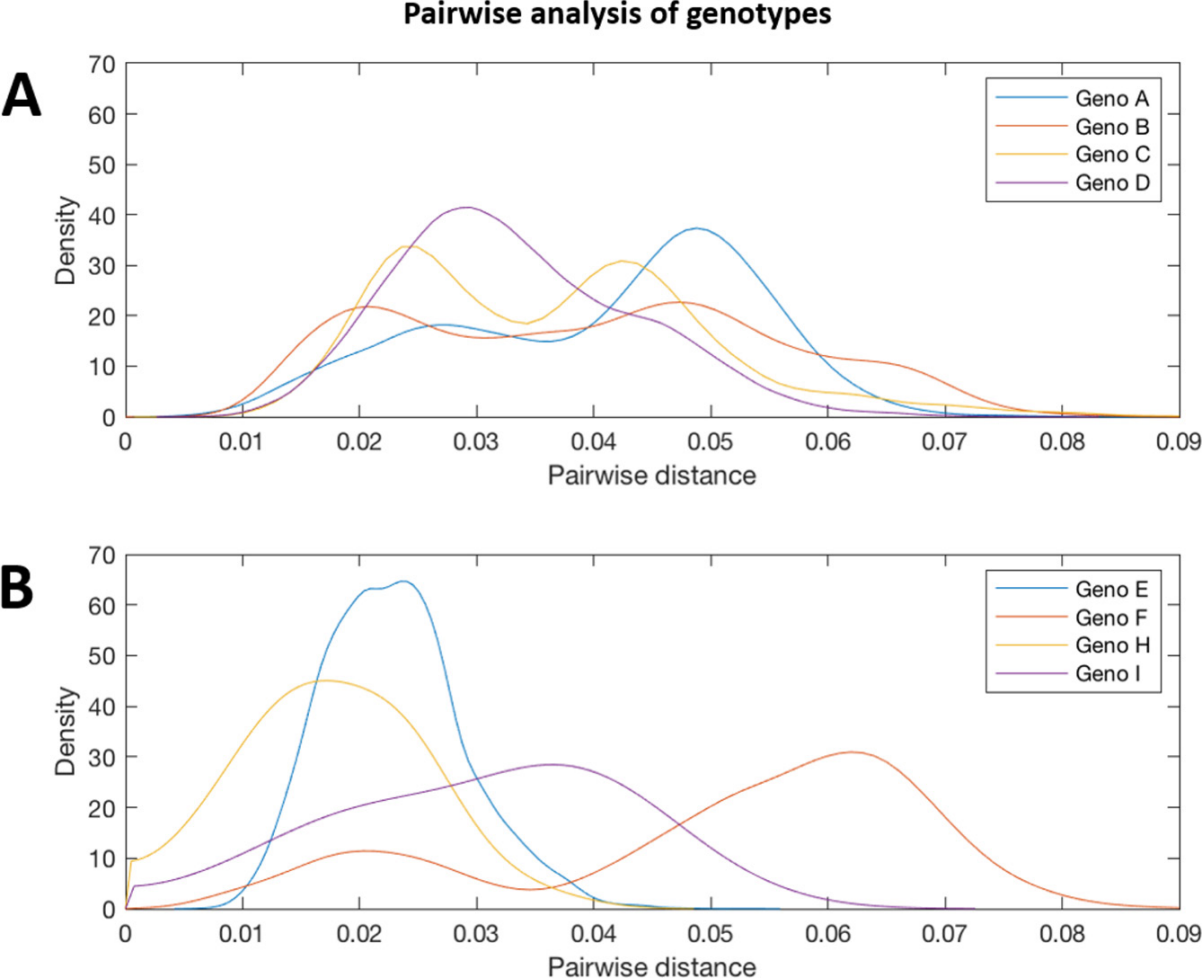
Pairwise distance distribution for the genomic-length sequences of HBV genotypes A, B, C, D, E, F, H and I. Probability densities of pairwise distances for whole-genome sequences of HBV genotypes. Genotypes E, F, H and I are shown on a separate plot from genotypes A–D as they contained smaller number of sequences. Too few sequences were available after filtering for genotypes G and (putative) genotype J to be analysed.

We aligned our newly defined genotype and subgenotype reference sequences and used them to generate a maximum-likelihood phylogenetic tree (Fig. 11). Pairwise distance analysis for the majority of genotypes (Fig. 12) revealed a bi-modal distribution of the distances, with one peak representing the relationship to sequences from the same (or closely related) clades (typically showing 1–3 % divergence) and the other peak being characteristic of distance to sequences in other clades from other subgenotypes (typically 3–8 % divergence). Genotype D differed from this distribution with a single peak where the majority of pairwise distances were between 2–4 %. This indicates that genotype D phylogeny does not contain long evolutionary branches separating clades from each other. Genotypes E, F, H and I contained smaller number of sequences, which can impact on the pairwise distance distribution. In this group genotypes E, H and I showed a uni-modal distribution of the pairwise distances. The lowest pairwise divergence was observed in genotype H, where the majority of pairwise distances were <3 %. Genotype F demonstrated the greatest between-sequence diversity, where a high proportion of pairwise distances were >5 %, likely a reflection of the long evolutionary distance between subgenotypes. Genotype G only had three sequences after our initial filtering, which was too small for a meaningful pairwise distance analysis.

## Discussion

### Summary of this resource

We have collated genomic-length HBV reference sequences into a repository (available on Figshare, https://doi.org/10.6084/m9.figshare.8851946), generating a resource that will help to underpin research. As clinical practice evolves to incorporate recommendations pertaining to HBV genotype, our dataset will also potentially become useful to support sequence-based insights for clinical practice. Many established databases are not currently designed for high-throughput work. Geno2pheno (https://hbv.geno2pheno.org/), for example, only allows four sequences at a time to be analysed, making progress slow with many sequences. This is the first resource to validate nomenclature to subgenotype resolution (removing subgenotypes for which there are inadequate data) at the same time as designating a unique reference sequence for each genotype and subgenotype. The reference sequences are provided in an accessible format that is compatible with both low- and high-throughput sequence analysis, making it useful for a variety of applications.

### Insights into diversity and geography

Genotype C has a particularly extensive phylogeny relative to the other genotypes. Genotype C is endemic in the Asia-Pacific region and is thought to be the oldest extant genotype [31]. The expansive diversity within this genotype is likely a consequence of its long evolutionary history in humans, although it should be noted that this genotype is also the best-represented genotype in the analysis, accounting for over 1/3 of all sequences in our dataset. Considerable diversity is also observed with genotypes B and D. Genotype D has a relatively broad geographical distribution, being found in regions including the Mediterranean, North-eastern Europe, India, Oceania and parts of southern Africa [32]. It is possible that persistence of chronic HBV infection in these varied ethnic groups has contributed to the diversification of this genotype.

Genotype F has an atypical phylogeny compared to the other genotypes, comprising four subgenotypes separated by long evolutionary distances, but with little intra-subgenotype diversity. It is unclear if the unusual phylogeny is a result of the elevated substitution rate documented for genotype F [33] or if there is a paucity of sampling relative to the other genotypes. The genotype is distributed throughout Latin America and the Arctic Circle [34, 35], regions that are under-represented by clinical and research cohorts to date. Genotypes E, G and H, each of which has a single subgenotype, have relatively constrained phylogenies, showing low levels of genetic diversity in the currently sampled isolates. Genotype G has a particularly low evolutionary rate, likely resulting from the 36 bp insertion in the core gene hampering viral replication [36]. The genotype is typically observed in co-infection with either HIV or HBV genotype A2 (36).

A HBV strain isolated from a 16th century skeleton (LT992440) clustered between subgenotypes in genotype A. The isolate falls (with long branch lengths) together with two sequences isolated from patients in Belgium, one of which has been proposed as the hypothetical subgenotype A6 (GQ331046), isolated in 2006 [37]. The clustering of these three sequences highlights the protracted association of HBV with human populations, and the marked lack of temporal population structure displayed by the viruses, frequently confounding phylogenetic attempts to understand the evolutionary history of HBV [22, 38].

### Limitations

#### Errors and inconsistencies in existing classification system

A number of previously described HBV subgenotypes were not identified using genomic-length HBV maximum-likelihood analysis in this study. A cause of these inconsistencies is the assigning of ‘provisional’ subgenotypes, based on a single isolate, as is the case with C7 and C9. Using the published literature to inform our analysis, we took an indication of strong bootstrap support, and a threshold of ≥2 sequences to define and identify the subgenotype clades. For some subgenotypes, including A4 and A6, multiple isolates have been reported in the literature, but few isolates were retained in our analysis after removing highly similar sequences, suggesting divergence within the subgenotype is limited. Despite this approach, a number of clades grouped distinctly away from known subgenotypes but could not be categorically assigned to a subgenotype (thus we designated two ‘unidentified clades’ in genotype C).

We suggest that, in future, a minimum number of epidemiologically unlinked sequences and genomic coverage (e.g. ≥2 genomic-length unlinked sequences) should be required to designate a new HBV subgenotype. Bias introduced into databases from intra-host diversity studies, where multiple sequences are isolated from the same patient (such as clonal analysis), can confound such analyses, but should have been largely controlled for in this study by stripping the alignments of similar sequences.

The phylogenetic methods selected may affect the subgenotypes classified, as there may be variability in bootstrap support between methods. Both neighbour-joining and parsimony methods are considered to be less accurate than maximum-likelihood approaches [39, 40], as used in this study. Recent work using Bayesian phylogenetic approaches to generate reference sequences for HBV was in agreement with our conclusions revising the number of subgenotypes downwards [41], illustrating the issue that several documented subgenotypes lack robust phylogenetic support. Future analysis to define HBV subgenotypes should therefore be based on maximum-likelihood methods and the bootstrap support should be indicated.

#### Bias in published data

There is considerable variation in the numbers of sequences available for each genotype, with genotypes B and C particularly well represented and genotypes E–I relatively neglected. Whilst these differences may reflect genuine variation in the contribution of different genotypes to the total global pool of infection – with B and C coming from high-prevalence, densely populated regions – significant undersampling of HBV sequences from other regions is likely (especially from low-income regions). Similar trends in isolate bias have been reported in the HCV field [42]. Consequently, the sequences available in publicly accessible databases are likely to under-represent the true extent of HBV diversity and, as more sequences are generated, reference sets will need to be reviewed.

Several previous studies publishing HBV reference sequence sets have generated consensus sequences from alignments of full-genome HBV sequences and submitted these new consensus sequences to GenBank [43, 44], which leads to the potential inference that these are sequences of biological origin. A number of genotype B, D and F sequences generated with the same approach have also been submitted to GenBank, but they are not supported by an associated publication, making it impossible to assess their provenance. The methods used to generate these sequences mean that, by definition, they occupy a basal position in the subgenotype clades after phylogenetic analysis and appear to be ideal reference sequences. However, these consensus sequences may differ from biologically derived sequences at key sites, and are therefore potentially misleading if used as reference sequences. Improved sequence metadata in the records of published sequences, clearly indicating the methods used to derive these sequences, would be informative for researchers.

#### Recombination and ‘quasi-subgenotypes’

Recombination is relatively common amongst HBV isolates [11, 45] and a number of established HBV subgenotypes have been shown to have a recombinant origin, particularly in genotypes A, C, D and the putative genotype J [45–47]. Recombination is likely to be even more prevalent within genotypes, however it is more difficult to detect between similar sequences. A number of sequences in our phylogenetic analysis were not easily classified, as they fall phylogenetically between two well-defined subgenotype clades. These sequences are possibly inter-subgenotype recombinants, combining genetic regions from two or more subgenotypes, making them challenging to classify in genomic-length phylogenies. Such recombinants are unlikely to have been filtered out by HBVdb [14], as the database only analyses sequences at the genotype level. Where possible, these sequences were excluded when defining the subgenotype clades to minimize their influence on the selection of reference sequences.

Previous work has indicated that some designated subgenotypes, including A3, A4, A5, C2 and B3, group as distinct, monophyletic clades in maximum-likelihood analysis, but that they do not meet the required genetic distance to be classified as separate subgenotypes [3, 9, 48]. It has been suggested these should instead be designated ‘quasi-subgenotypes’ to reflect their divergent nature but underscore that they do not meet the technical definition of a subgenotype [9]. In our analysis, quasi-subgenotype A3 grouped into A3, A4 and A5 clades with strong bootstrap support. It was not possible to separate subgenotypes C2 and quasi-B3 (encompasses B3 and B5) into distinct subgenotypes.

### Conclusion

Sequencing is increasingly utilized in the clinical management of HBV infection to inform on prognosis, treatment choice and drug resistance and to provide insight into complex cases [49, 50]. Whilst progress in applying new sequencing technologies to HBV has been slow to date, we are now entering an era of rapid change. A consistent, unified approach to classification will advance this field, improving the consistency with which sequence data are curated, archived and reported. Recent work sampling poorly accessed populations has uncovered numerous new HCV isolates [51, 52], and the diversity of sequences generated for HBV may expand in a similar way. The use of a robust and consistent classification system will also be important in screening new populations for potentially novel HBV isolates. The resource generated in this study is versatile and suitable for both low- and high-throughput sequence analysis. All of the generated data, including the alignments, phylogenies, consensus sequences and chosen reference sequences (Fig. 11), are available online as a simple open-access resource (https://doi.org/10.6084/m9.figshare.8851946). We have generated a new data resource for researchers and clinicians in the HBV field, providing a solid foundation for analysis, and a structure on which to build as more data emerge.

## Supplementary Data

Supplementary material 1
