# Supplementary material for: Analysis of genomic-length HBV sequences to determine genotype and subgenotype reference sequences
Source: J Gen Virol. 2020 Mar 5;101(3):271–83. doi: 10.1099/jgv.0.001387 (PMC7416611; doi:10.1099/jgv.0.001387)

Supplementary Material

Analysis of full-length HBV sequences to determine genotype and subtype reference sequences: a data resource to support research and clinical practice

Anna L McNaughton, Peter A Revill, Margaret Littlejohn,
Philippa C Matthews, M Azim Ansari

Supplementary figure 1; **Cumulative number of (A) all HBV sequences and (B) whole genome HBV sequences in Genbank, 2000-2018.** Data downloaded directly from Genbank in December 2019.

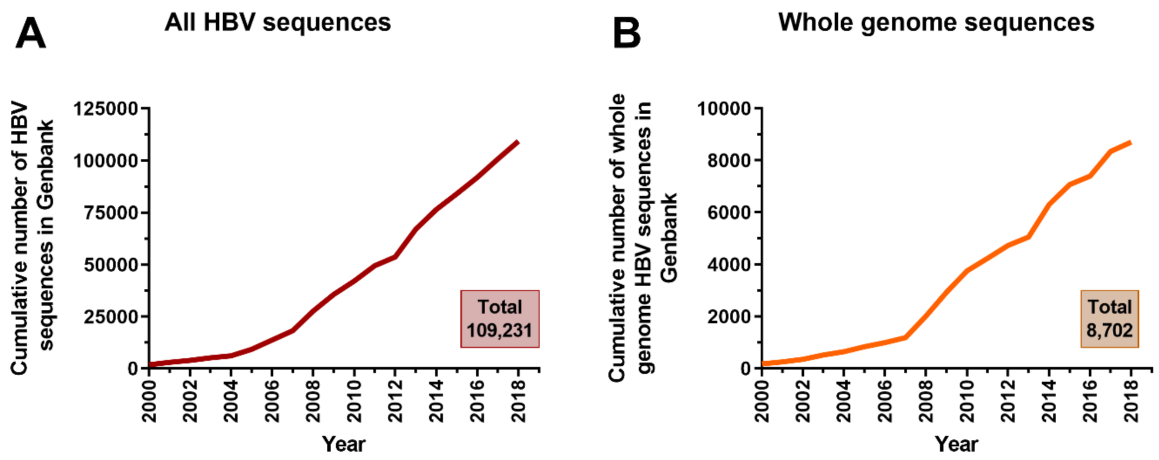

Supplement: Supplementary material 1 [file jgv-101-271-s001.pdf]
